# Virtual Reality Therapy Assistants for Adjunctive Cue Exposure Training in the Treatment of Alcohol Use Disorder (VITAACue-AUD): Protocol for a Technical Feasibility Study

**DOI:** 10.2196/99080

**Published:** 2026-10-20

**Authors:** Kornelius I Kammler-Sücker, Carolin Heizmann, Katharina Eidenmueller, Leon Werner, Thomas Prexl, Frank Rust, Rolf-Dieter Schiermeyer, Melissa R M Mohr, Philipp Kellmeyer, Bernd Lenz

**Affiliations:** 1 Center for Innovative Psychiatry and Psychotherapy Research, Central Institute of Mental Health, J5, Mannheim, Baden-Wurttemberg, 68159, Germany, 49 17036359; 2 Partner Site Mannheim-Heidelberg-Ulm, Deutsches Zentrum für Psychische Gesundheit, Mannheim/Heidelberg/Ulm, Baden-Wurttemberg, Germany; 3 Data and Web Science Group, School of Business Informatics and Mathematics, University of Mannheim, Mannheim, Baden-Wurttemberg, Germany; 4 Human-Technology Interaction Lab, Department of Neurosurgery, University Medical Center Freiburg, Freiburg, Baden-Wurttemberg, Germany; 5 Department of Addictive Behavior and Addiction Medicine, Central Institute of Mental HealthMannheim, Baden-Wurttemberg, Germany; 6 neunzehn innovations GmbH, Heidelberg, Baden-Wurttemberg, Germany

**Keywords:** alcohol use disorder, virtual reality, large language model, generative artificial intelligence, cue exposure, feasibility study, digital mental health, addiction treatment, participatory design

## Abstract

**Background:**

Alcohol use disorder (AUD) is associated with a substantial individual and societal burden. Cue exposure approaches can support treatment by targeting conditioned cue reactivity and high-risk contextual associations. However, routine implementation is limited by therapist time, scalability constraints, and the difficulty of reproducing ecologically valid drinking situations in standard care settings. Virtual reality (VR) and AI have been proposed as promising tools to support scalable and contextually rich interventions in mental health care.

**Objective:**

This study aims to develop and technically evaluate a prototype virtual reality–based therapy assistant (VRTA) for narratively guided alcohol cue exposure in immersive VR.

**Methods:**

This is a single-site technical feasibility study conducted at the Central Institute of Mental Health in Mannheim, Germany. It builds on a participatory development process, including multistakeholder workshops with developers, researchers, clinicians, and patient and public involvement contributors. The study began with workshops to refine implementation priorities. A VRTA prototype will be developed to guide alcohol cue exposure scenarios through speech-based interaction with a computer-generated character in immersive VR, informed by established exposure manuals (development ongoing, currently with a text-based chatbot prototype and a separate VR environment). An iterative qualitative expert appraisal will be conducted during development (ongoing). In addition, 20 adult patients with AUD will participate in an observer-based evaluation in which therapeutic staff demonstrate the prototype and patients provide qualitative feedback.

**Results:**

Results are expected on the technical feasibility of the VRTA prototype, including interaction quality and integration into therapeutic workflows. Qualitative findings from expert appraisal and patient feedback (observed from a third-person perspective) will provide insights into usability, manual conformity, acceptability, and perceived therapeutic relevance (audio transcript coding and qualitative evaluation are ongoing). Funding for this study has been approved by the Hetzler Foundation from November 2025 to October 2027. Initial workshops were conducted in January 2026 and May 2026. Prototype testing by expert participants with patient observers is planned to start in November 2026 and continue until April 2027.

**Conclusions:**

This study is intended to generate design specifications and feasibility data to inform subsequent prototype refinement and future clinical evaluation studies.

## Introduction

Alcohol use disorder (AUD) remains a major contributor to global morbidity and mortality and is characterized by complex interactions between neurobiological, psychological, and environmental factors. A central challenge in AUD treatment is the high rate of relapse, which is strongly influenced by conditioned responses to alcohol-related cues and contexts [1,2]. These conditioned responses can trigger craving and habitual drinking behavior, even after prolonged periods of abstinence.

Cue exposure therapy aims to reduce such conditioned responses through repeated and controlled exposure to alcohol-related stimuli without subsequent consumption. A recent meta-analysis suggests that cue exposure therapy can be an effective adjunctive intervention in AUD treatment, although effect sizes vary and implementation remains inconsistent [3]. One of the main barriers to widespread adoption is the difficulty of reproducing ecologically valid high-risk situations within clinical settings. Traditional exposure approaches often rely on imagination or limited physical cues, which may not adequately capture the complexity of real-world environments.

Virtual reality (VR) offers a promising solution to this limitation by enabling immersive, controllable, and contextually rich simulations of real-life situations. VR immerses users in a computer-generated environment and elicits strong behavioral responses via a sense of presence [4], of “being there” and of really interacting with virtual objects in a plausible world [5,6]. VR has been proposed as a powerful tool to investigate and potentially enhance treatment mechanisms in addiction by providing realistic exposure environments while maintaining experimental control [1,7-9]. In addition, VR allows repeated and standardized exposure sessions without requiring extensive therapist resources.

At the same time, advances in digital psychiatry have highlighted the potential of AI-based systems, including conversational agents, to support mental health interventions [10-12]. Large language models (LLMs) enable flexible and context-sensitive interaction, which may be particularly relevant for guiding therapeutic processes such as exposure exercises. However, these technologies also raise important concerns related to reliability, transparency, and alignment with clinical standards, especially in sensitive applications involving vulnerable populations [13,14].

Narratively guided cue exposure is a particularly suitable use case for combining VR and AI, as the therapeutic interaction can be structured according to established treatment principles and manuals [15,16]. This allows for the development of systems that operate within defined therapeutic boundaries while still enabling flexible interaction.

In the development of digital health applications, early integration of expertise across the development process is increasingly important. Not only are scientific and clinical experts involved in the early stages of technological prototype development but other stakeholders involved in the intended therapeutic application are also increasingly being included. These include, in particular, “experts-by-experience”—that is, current or former patients. Various forms of patient and public involvement (PPI) are routinely used in the development processes of health applications [17,18]. They allow for the inclusion of first-person knowledge of patients’ lived experiences in health research [19] and in the development of interventions in addiction [19,20]. For the technical development of health-related applications, this includes, in particular, the observations of experts-by-experience during prototype testing and thus promises to both foster clinical utility and real-world usability for patient users. One possibility is the integration of experts-by-experience and other stakeholders into regular co-design workshops, where design choices and current development stages are discussed and tested in a nonhierarchical group setting. Audio recordings of such workshops can also be analyzed afterward with qualitative methods to summarize and organize key points and insights that emerged during the workshops [21,22]. Such approaches are increasingly used for chatbot applications for mental health [23] as well as for health-related VR setups [24].

The present study focuses on the development and technical feasibility evaluation of a VR-based therapy assistant (VRTA) designed to guide alcohol cue exposure scenarios. The study follows a participatory development approach [22]. Two preliminary multistakeholder workshops, involving developers, researchers, clinicians, and PPI contributors, were held in January and May 2026 to identify key design requirements and potential challenges (coding of audio transcripts and qualitative evaluation are ongoing). Building on this preparatory work, the study will refine implementation priorities and evaluation criteria for the feasibility phase.

## Methods

### Study Design

This study is a single-site, prospective, qualitative technical feasibility study conducted at the Central Institute of Mental Health in Mannheim, Germany. The study started in January 2026 and will continue until the end of 2027.

The study is structured as an iterative development and evaluation process consisting of 3 main components: an initial participatory workshop phase, ongoing prototype development with expert evaluation, and an observer-based patient appraisal.

### Participatory Development Approach

The study is embedded in a participatory design framework. Prior to study initiation, a multistakeholder workshop was conducted in January 2026, involving developers, researchers, clinicians, and PPI contributors, in this case, experts-by-experience who were former patients with AUD. This workshop identified key requirements, potential risks, and design priorities for the VRTA system.

A second workshop was held in May 2026 with a similar stakeholder composition. The workshop was structured as a facilitated co-design session combining plenary discussions and small-group formats. Building on the initial workshop, participants refined key system components such as the narrative structure of exposure scenarios, interaction modalities, and integration into therapeutic workflows, while also identifying potential risks and boundary conditions for safe system behavior.

Both workshops were audio-recorded after having obtained informed consent from all participants. Qualitative analyses of these recordings will be published elsewhere. On the basis of these findings, evaluation criteria for the feasibility phase were defined, including technical performance, interaction quality, usability, acceptability, and alignment with therapeutic manuals. Stakeholders also helped identify relevant outcome domains and qualitative indicators for subsequent expert and patient-based evaluations. The outputs, that is, refined design specifications and a preliminary evaluation framework, will directly inform prototype development and iterative testing.

### Prototype Development

The VRTA prototype is designed to guide narratively structured alcohol cue exposure scenarios within immersive VR environments. The intended system integrates VR-based simulations of alcohol-related contexts with speech-based interaction facilitated by a LLM. The virtual agent responds to user input and provides guidance consistent with exposure-based therapeutic principles.

The development process will explore different technical configurations, including the use of secure, institutionally hosted servers compliant with relevant data protection standards. The feasibility of real-time processing and interaction will be a central focus of the study.

In addition to the intended fully integrated system architecture, an intermediate implementation strategy is planned to ensure feasibility in case of technical or infrastructural constraints. This approach involves decoupling the immersive VR exposure environment from the LLM-based narrative guidance component. In this intermediate configuration, a VR-based alcohol cue exposure setup will be deployed as a standalone system to provide immersive visual and contextual stimuli. In parallel, the LLM-based narrative guidance will run on a separate machine, enabling speech- or text-based interaction independent of the VR system. The 2 components will be coordinated at the level of the experimental or therapeutic procedure rather than through full technical integration.

This approach allows evaluation of the core functional elements of the VRTA concept—namely, immersive cue exposure and adaptive narrative guidance—even if real-time integration of both components within a single system is not technically feasible at early stages of development. It also enables staged testing of interaction quality, timing, and user experience while maintaining system architecture flexibility.

If full integration proves feasible, the intermediate setup will be replaced by a unified system combining VR and LLM-based interaction. If not, the decoupled configuration may serve as a fallback solution for further development and evaluation. This staged implementation strategy is intended to reduce technical risk while preserving the ability to assess key aspects of the proposed intervention.

### Current Prototype Architecture

The current prototype design captures a stage within the iterative development process. Thus, most technical details reported in the following are still potentially open to considerable change, depending on technical requirements and critical feedback from clinical experts and experts-by-experience, the latter in their role as patient observers. The current stage of the prototype consists of a chatbot interface displayed in a typical chat-like manner on a 2D screen, with interactions based either on text entry or on human users’ verbal communication via speech-to-text recognition, and with text-to-speech output from the VRTA. The current version of the *chatbot prototype* uses GPT-5.1 (OpenAI), with instances on European servers accessed online. However, future versions may switch to different LLMs. Beyond the scope of this study, future real-world interactions with patients or experts-by-experience are planned to rely on LLM instances running on data-secure, nonprofit servers within the European Union (eg, German academic servers) or, if possible, locally on suitably equipped computers. The chatbot is prompted to act as a therapeutic assistant guiding a patient through an alcohol cue exposure session in VR; it is instructed to appeal to participants’ imagination to evoke typical situations and contexts of alcohol consumption (eg, by asking what would typically have happened before such a situation) to further intensify the audiovisual VR environment by adding personalized imaginative details. The chatbot is also instructed to guide participants through virtual interactions with a glass, a bottle, and a beverage, and to help them observe their own perceptual, affective, physiological, and cognitive responses and to track them over time during the exposure. It regularly asks them about their current craving on a numerical scale. The dialogue is organized as a sequence of exposure phases implemented through phase-specific prompt components. After each user interaction, the system estimates from the conversation history whether the current phase should continue or whether the next phase should be initiated. Depending on this estimate, the chatbot’s prompt context is updated with the corresponding phase instructions, such as “introduction,” “attending to the closed bottle,” “opening the bottle,” “pouring the beverage into a glass,” “approaching the glass,” and “closing the session.” Thus, the prototype does not rely on a single static instruction prompt but iteratively adapts the prompt basis to the current stage of the exposure dialogue. In all of these aspects, strict manual adherence of the chatbot is supported by detailed prompt instructions based on commonly used instructive materials on alcohol cue exposure for psychiatrists and psychotherapists. Accordingly, the chatbot is intended to politely direct users back to their current task, to stay within the situation and observe their perceptions, feelings, and thoughts therein, even if users try to deflect the conversation or engage the VRTA in discussions about the procedure from a distant point of view. If users want to quit, the VRTA encourages them to stay in the exposure session, as a therapist would, yet will respond to signals of severe stress, such as suicidality, by closing the session in an orderly manner and referring users to the human therapist who is present nearby. Note that at this stage, the situation is simulated, as no actual patient will interact with the setup, yet the chatbot is instructed to act “under the pretense” of this situation.

The current version of the *VR environment* is based on developments from earlier studies [7,9] and displays different virtual environments (eg, a home flat and a pub) that allow for the virtual inspection of alcoholic beverages in bottles and their pouring into a glass. The current setup runs on a gaming notebook (GPU: NVIDIA GeForce RTX 4090) connected to a Meta Quest 3 in tethered (cable-bound) mode, with virtual environments developed and compiled in Unity3D (Unity Technologies).

The current *combination design* sets up chatbot interaction and a VR environment on 2 separate machines without communication between them. The chatbot VRTA will guide expert participants via an audio signal while they are immersed in and engaging with the virtual environment. Meanwhile, patient observers will be able to observe a 2D screen projection of the environment. On the basis of findings from the initiating stakeholder workshops, the current plan is to retain the “disembodied” character of the VRTA voice even when the chatbot and VR would run on the same machine and to potentially give it a slightly alienated, rather “machine-like” tone. These aspects came up during workshop discussions among experts-by-experience. On the one hand, these discussions clarified that a virtually embodied social presence, even in the form of a virtual character embodying a nonhuman VRTA, may attenuate the virtual scenery of “sitting alone in front of the glass,” and hence serves as a welcome distraction from the most challenging aspects of the exposure situation. On the other hand, a context of virtual solitude was deemed to most closely resemble the most challenging situations in terms of potential relapse for most patients with AUD. Hence, the current design plan aims to disturb virtual seclusion as little as possible and will focus on a disembodied chatbot voice with rather “mechanical” inflection.

### Participants

Three groups of contributors will be involved in the study: expert participants, that is, clinically active therapists and scientific staff, as well as experts-by-experience, former patients in AUD treatment affiliated with the study site, and patient observers. Only expert participants will operate the prototype. These expert participants will directly interact with the prototype during development and provide qualitative feedback on its functionality, usability, and clinical plausibility. Patient observers will consist of 20 adults diagnosed with AUD according to the *DSM-5* (*Diagnostic and Statistical Manual of Mental Disorders, Fifth Edition*), aged between 18 and 64 years, who can provide informed consent. Patients will be recruited through clinical services at the study site. Observations of patient observers and the verbal interaction of expert participants with the VRTA will be audio-recorded, after obtaining prior informed consent from all parties. This sample size has been chosen to obtain a dataset suitable for meaningful qualitative analysis and, for pragmatic reasons, to remain within the limited time and resources available during the development process.

### Procedures

During the iterative development phase, expert participants will test different versions of the prototype and provide qualitative feedback. This feedback will be used to refine the system in successive development cycles.

Patient participants will take part in an observer-based evaluation. In these sessions, a trained clinician will demonstrate the use of the VRTA prototype in a simulated therapeutic scenario. Patients will observe this demonstration and subsequently provide an assessment of the system’s plausibility, usability, and acceptability. Patients will not directly interact with the prototype. This is due to the sensitive nature of the setup; in these early stages of development, further adversarial testing and piloting with participant experts should ensure reliable assessments of safety and guardrails before establishing direct patient interactions with the system.

The iterative evaluation of the prototype will be conducted by technical, scientific, and clinical experts in an ongoing process, with additional input from patient observers. This process involves usability and feasibility testing in simulated interactions as they typically occur in the clinical practice of alcohol cue exposure. For this purpose, nonpatient participants will play fictional patient personas in their simulated interactions. This type of testing already involves considerable strain on the chatbot’s communicative guardrails, as alcohol cue exposure interactions may prompt patients to avoid or escape the situation by deflecting the verbal interaction away from the exposure scenario. In addition to this type of testing, technical, scientific, and clinical experts engage in thorough adversarial testing throughout prototype development, which involves, among other approaches, attempts at prompt injection and hostile interactions with the chatbot; these human behaviors are not actually expected for most real-world interactions with patients in potential future settings, as patients undergoing exposure therapy have typically already displayed a high level of compliance and therapy adherence before entering this stage of their therapeutic process. However, testing these extreme boundary cases will help ensure comprehensive patient safety in future applications.

As this is a noncomparative, early-phase technical feasibility study focused on prototype development, no control condition is included.

### Outcomes

The primary outcomes of the study are qualitative assessments of technical and experiential feasibility. These include the feasibility of implementing the VRTA system, the feasibility of real-time interaction under secure data-processing conditions, and the perceived realism, immersion, usability, clinical plausibility, and manual conformity of the system.

These outcomes will be assessed by qualitative interviews with expert participants and patient observers. Structured interviews will give room for new aspects and observations of interviewees to emerge, while also consistently addressing predefined questions. These questions will initially focus on interviewees’ impressions of technical aspects, including latencies, intuitive interaction with the chatbot and the environment, simplicity of use, presence, and social copresence (also assessed with the iGroup Presence Questionnaire [5]), with the latter points only assessed by expert participants who experience the VR from a first-person perspective. They will then address clinical plausibility, including how closely the session would resemble real-world exposure in terms of sensory stimulation and both physical and social context; how viable interviewees deem the practical use of the system in clinical practice; how likely interviewees consider patients to be to engage with the challenging exposure situation in such a setup; and how plausible they deem the clinical realism for patients, that is, the “successful” elicitation of craving-related symptoms and negative affect that are to be addressed in exposure therapy. Finally, interviews will address concerns of guardrailing, safety, and manual conformity, with interviewees giving their assessments of dangers of prompt injections by users and inappropriate (eg, adversarial communication by the VRTA); of challenged users successfully leading the VRTA “off the rails” from the predefined path of exposure therapy sessions as defined in therapy manuals; and of potential concerns regarding data security with respect to potentially sensitive personal data of future users.

In all of these domains, predefined criteria will guide whether the prototype is sufficiently mature to progress to direct patient testing. Technical feasibility and real-time interaction will be assessed based on system failures, latencies, and interruptions; realism and usability through interviews and presence ratings; and clinical plausibility through expert assessment of consistency with real-world therapeutic practice. Manual conformity and safety will be measured against strict criteria before proceeding to preclinical testing, with protocol deviations, inappropriate responses, successful attempts to steer the VRTA outside its intended scope, and data security concerns systematically documented and eliminated through further adjustments. Progression will require resolution of all safety-critical issues and absence of unresolved deviations judged to pose clinically meaningful risk, while noncritical limitations may inform further iterative refinement.

### Data Collection and Analysis

Data will be collected through qualitative feedback from expert participants and patient observers. Patient data will be collected anonymously, and no personally identifiable information will be recorded. The workshop sessions will be audio-recorded if written informed consent is provided and supplemented by field notes to document contextual observations. Recordings will be automatically pretranscribed using aTrain [25] and subsequently reviewed, corrected, and deidentified by the research team. In addition to transcripts, field notes and workshop materials will be used to contextualize and enrich the data. All qualitative data will be coded in MAXQDA (VERBI GmbH) [26] and analyzed using qualitative content analysis [27], with a focus on systematic coding and category development to identify strengths, limitations, usability concerns, perceived clinical plausibility, and areas for improvement. Findings will be used to refine the prototype iteratively and to inform future feasibility and evaluation studies.

### Ethical Considerations

The study was approved by the Ethics Committee II of the Medical Faculty of Heidelberg University (2025‐655). It will be conducted in accordance with applicable ethical guidelines and data protection regulations, including the General Data Protection Regulation. The study is not registered in a clinical trials registry. All participants will receive detailed written and verbal information about the study objectives, procedures, and data handling prior to participation. Written informed consent will be obtained before any study-related procedures are conducted, including consent for audio recording. Participation is voluntary, and participants may withdraw from the study at any time without providing a reason and without any negative consequences. Given the involvement of individuals with (former) AUD and the use of immersive VR environments, particular attention will be paid to participant safety and well-being. Exposure-related content will be embedded within a controlled research setting, conducted exclusively in a supervised clinical environment rather than in participants’ homes, and study procedures will be conducted in the presence of clinically trained personnel, including psychiatrists and psychologists involved in the study. During all study procedures, participants will be monitored, and appropriate measures will be taken to ensure that no excessive psychological burden arises while acknowledging that exposure procedures may induce transient emotional or craving-related responses as part of the therapeutic process. Potential VR-related physical discomfort (eg, dizziness or cybersickness) will be monitored and addressed as needed. If necessary, study participants will receive immediate access to professional clinical support and monitoring. All data will be handled in deidentified form. Audio recordings and transcripts will be stored on secure servers, and access will be restricted to authorized members of the research team. The system operates within predefined therapeutic guidelines to ensure clinically appropriate interactions. No personally identifiable information will be included in the analysis or dissemination of results. The LLM component is accessed through a European-hosted API (current stage of prototype development); only text prompts generated by trained clinical and research staff are transmitted, while patient observers neither interact with the system nor provide data to it. Entry of identifiable information or sensitive personal or health data is prohibited, and no patient audio or other patient-derived data are transmitted to the external LLM service. All transmitted content is restricted to nonidentifiable, nonsensitive information generated within the simulated research setting. Further development aims to implement fully data-secure solutions via use of institutionally hosted servers or, where technically feasible, entirely local LLM processing.

## Results

The study is expected to generate information on the technical feasibility of the VRTA prototype, including interaction quality and integration into therapeutic workflows. Qualitative findings from expert appraisal and patient feedback will provide insights into usability, acceptability, and perceived therapeutic relevance.

Funding for this study has been approved by the Hetzler Foundation from November 2025 to October 2027. Initial workshops were conducted in January 2026 and May 2026. Prototype testing by expert participants with patient observers is planned to start in November 2026 and continue until April 2027.

In the course of this study, a comprehensive dataset comprising recordings of workshop discussions and testing sessions, field notes by study personnel, and posttesting interviews with expert participants and patient observers will be acquired and analyzed using qualitative methods (coding of audio transcripts and qualitative evaluation are ongoing). The study is expected to yield qualitative insights into challenges and facilitating factors specific to the development of a VRTA for a highly standardized psychotherapeutic procedure in AUD at this prototype stage, including aspects of natural language interaction between users and the technical system. The qualitative analyses will identify recommendations for future design development and highlight critical points for securing safety, usability, and clinical utility. These insights will be aggregated and structured by qualitative analysis, which can later inform both future design development and the construction of potential quantitative assessment methods for VRTA setups in AUD treatment.

## Discussion

### Anticipated Findings

This project aims to develop a prototype for a VRTA for narratively guided alcohol cue exposure in immersive VR. It uses a participatory design process already in early stages of development, thereby involving technical, scientific, and clinical experts as well as experts-by-experience (former patients as participants in co-design workshops, current patients as observers of prototype testing by expert participants). The project has 3 main outcome goals: first, a workable prototype ready for quantitative testing and eventual direct patient interaction in future studies; second, insights into the current level of safety, comfort, usability, and feasibility of the prototype; and third, insights into design specifications to fulfill requirements of all stakeholders in future development stages, and a list of suitable assessment metrics to check for safety (including cybersickness), practical usability and comfort, and clinical efficacy and utility.

This study represents an early-stage investigation into the feasibility of combining immersive VR and AI-based interaction to support cue exposure in AUD. By focusing on technical feasibility and qualitative evaluation, the study aims to establish a foundation for subsequent research rather than to assess clinical efficacy. To inform prototype development with first-person-perspective insights from the start, experts-by-experience are included as observers to share their advice and insights during this development, even before extensive prototype testing for quality assurance (as planned for future follow-up studies), which allows for ethically responsible testing of direct patient interactions with the prototype.

On the basis of prior work, integrating VR and AI has the potential to address key limitations of traditional cue exposure approaches. Whether this potential can be realized in the current VRTA prototype is a central question addressed by the current study. However, the use of AI in therapeutic settings raises important challenges related to reliability, transparency, and trust that must be carefully addressed during development [14,28]. In addition, eliciting social copresence with a VRTA, that is, the feeling of “being with another person,” may be a double-edged sword in alcohol cue exposure, due to a potential trade-off between user engagement and anthropomorphism. A parasocial communicative bond with the VRTA may motivate greater perseverance and adherence during exposure, similar to the psychological support offered by AI agents in other settings [12]. The potential downsides of this can be tendencies to anthropomorphize the VRTA [29], which may foster experiential avoidance among participants during exposure by drawing their attention toward the (virtual) social presence of a VRTA. This may be exacerbated by tendencies of LLM agents to simulate communication with higher levels of compassion and empathy than human counterparts [30] and to overly confirm and reinforce any verbal expressions of human users, a phenomenon that can be termed overvalidation [31] or sycophancy [32]. Thus, the complex effects of perceived social copresence have to be carefully considered in the current design, as in VR setups in general [33]. A successful prototype design should also be assessed by how well it averts such tendencies through the described efforts to ensure the chatbot’s adherence to its therapy manual and to guard against overvalidation of users’ inputs.

A key strength of this study is its participatory design approach, which incorporates input from multiple stakeholder groups, including PPI contributors. This approach is expected to enhance the relevance, usability, and acceptability of the developed system. This is especially important for the sensitive field of application to which LLM-based chatbots are introduced when used to assist psychotherapy. The already intricate and complex social dynamics of psychotherapeutic settings are thereby extended by the inclusion of an “artificial third” party [34]. Thus, it may be especially fruitful here to include different stakeholders in the design process of such frameworks early on, within a co-design setting where they can discuss and exchange their perspectives.

The study also has several limitations. It is conducted at a single site and involves a relatively small sample of patient participants. Furthermore, patients do not directly interact with the system, which limits the ability to assess user experience in real-world use. Finally, the study does not include quantitative or clinical outcome measures and therefore cannot provide evidence regarding treatment effectiveness.

Future research should build on this study’s findings to conduct controlled evaluations of the VRTA system, including direct patient interaction and assessment of clinical outcomes. Such studies will be necessary to determine whether the system can contribute to improved treatment outcomes in AUD.

We will disseminate the findings of this study through peer-reviewed journal articles and presentations at national and international conferences in psychiatry, addiction medicine, and digital health. The results will be shared with participating stakeholders, including clinicians, technical experts, and experts-by-experience, through institutional seminars and stakeholder briefings to support translation into practice and further development. Finally, key insights regarding the usability, safety, and perceived therapeutic relevance of the VR therapy assistant will be communicated to patient and public audiences in accessible formats, such as lay summaries.

### Conclusions

VITAACue-AUD (Virtual Reality Therapy Assistants for Adjunctive Cue Exposure Training in the Treatment of Alcohol Use Disorder) is a technical feasibility study of an LLM-supported VRTA for narratively guided alcohol cue exposure in AUD. By prioritizing technical implementation, prototype behavior informed by manual input, participatory design, and qualitative stakeholder appraisal before any direct patient application, the study is designed to generate the evidence needed to inform a decision on whether this concept warrants progression to later-stage studies involving direct patient interaction and, ultimately, clinical efficacy testing.

## Abbreviations

AUD: alcohol use disorder
*DSM-5*: *Diagnostic and Statistical Manual of Mental Disorders*, *Fifth Edition*
LLM: large language model
PPI: patient and public involvement
VITAACue-AUD: Virtual Reality Therapy Assistants for Adjunctive Cue Exposure Training in the Treatment of Alcohol Use Disorder
VR: virtual reality
VRTA: virtual reality–based therapy assistant
